# *Helicobacter pylori* CagA-mediated ether lipid biosynthesis promotes ferroptosis susceptibility in gastric cancer

**DOI:** 10.1038/s12276-024-01167-5

**Published:** 2024-02-21

**Authors:** Yanmei Peng, Xuetao Lei, Qingbin Yang, Guofan Zhang, Sixiao He, Minghao Wang, Ruoyu Ling, Boyang Zheng, Jiayong He, Xinhua Chen, Fengping Li, Qiming Zhou, Liying Zhao, Gengtai Ye, Guoxin Li

**Affiliations:** 1 Department of General Surgery & Guangdong Provincial Key Laboratory of Precision Medicine for Gastrointestinal Tumor, Nanfang Hospital, The First School of Clinical Medicine, Southern Medical University, Guangzhou, Guangdong 510515 China; 2 School of Medicine, The Chinese University of Hong Kong, Shenzhen, 518172 China

**Keywords:** Targeted therapies, Cell death

## Abstract

*Helicobacter pylori*, particularly cytotoxin-associated gene A (CagA)-positive strains, plays a key role in the progression of gastric cancer (GC). Ferroptosis, associated with lethal lipid peroxidation, has emerged to play an important role in malignant and infectious diseases, but the role of CagA in ferroptosis in cancer cells has not been determined. Here, we report that CagA confers GC cells sensitivity to ferroptosis both in vitro and in vivo. Mechanistically, CagA promotes the synthesis of polyunsaturated ether phospholipids (PUFA-ePLs), which is mediated by increased expression of alkylglycerone phosphate synthase (*AGPS*) and 1-acylglycerol-3-phosphate O-acyltransferase 3 (*AGPAT3*), leading to susceptibility to ferroptosis. This susceptibility is mediated by activation of the MEK/ERK/SRF pathway. SRF is a crucial transcription factor that increases *AGPS* transcription by binding to the *AGPS* promoter region. Moreover, the results demonstrated that CagA-positive cells are more sensitive to apatinib than are CagA-negative cells, suggesting that detecting the *H. pylori* CagA status may aid patient stratification for treatment with apatinib.

## Unlocking ferroptosis: CagA’s role in gastric cancer cells

Gastric cancer, a major cause of cancer death globally, is often associated with chronic infection by a bacterium named Helicobacter pylori. A specific strain of H. pylori that produces a protein named CagA is particularly harmful, as it can cause severe stomach diseases, including cancer. However, the connection between CagA and a kind of cell death known as ferroptosis in gastric cancer cells was unclear. In this research, scientists discovered that CagA boosts the production of certain fats in cells, making them more prone to ferroptosis. The study was an experiment involving both cell cultures and animal models. The researchers concluded that targeting the pathway influenced by CagA could be a new strategy for treating gastric cancer, especially in patients infected with CagA-positive H. pylori strains.

This summary was initially drafted using artificial intelligence, then revised and fact-checked by the author.

## Introduction

Gastric cancer (GC) is the fifth most commonly diagnosed cancer and the fourth leading cause of cancer death, with more than one million new cases and more than 700,000 deaths per year^1^. Chronic infection with *H. pylori*, particularly strains that possess cytotoxin-associated gene A (CagA), plays a key role in the development of gastric cancer^2^. CagA, a 128–145 kDa protein encoded by the 40 kb cag-pathogenicity island (cag PAI) of *H. pylori*, has been described as the first bacterial oncoprotein^3^. The CagA protein has been recognized as a marker of the entire cag island and has an important function. CagA has been shown to interact with approximately 25 host cell signaling factors and cause a wide array of cellular alterations, including inflammation, increased proliferation, genetic instability, tight junction alterations, dephosphorylation of cellular proteins, and chemoresistance^4–9^.

Ferroptosis, an iron-dependent form of nonapoptotic cell death that is associated with lethal lipid peroxidation, can be inhibited by iron chelators and antioxidants^10,11^. Lipid peroxidation is a free radical-driven reaction that mainly affects unsaturated fatty acids in cell membranes^12^. Recent studies have shown that polyunsaturated ether phospholipids (which include alkyl-acylphospholipids and alkenyl-acylphospholipids, also known as plasmalogens) act as substrates for lipid peroxidation and promote susceptibility to ferroptosis^13^. Ether phospholipids are an important group of phospholipids containing an ether linkage at the glycerol sn-1 position and usually polyunsaturated fatty acid (PUFA) at the sn-2 position^14^. The acquisition of a high-PUFA-ePL state promoted by upregulating the expression of ePL biosynthetic enzymes, such as AGPS (alkylglycerone phosphate synthase) and AGPAT3 (1 acylglycerol-3-phosphate O-acyltransferase 3), can increase susceptibility to ferroptosis^13^. Emerging evidence indicates that the activation of ferroptosis is implicated in tumor suppression^15^. In addition, ferroptosis has been detected in many infectious diseases, such as hepatitis C, severe acute respiratory syndrome coronavirus 2 (SARS-CoV-2) infections, *Mycobacterium tuberculosis* infections, and *Pseudomonas aeruginosa* infections^16–19^. However, the link between *H. pylori* CagA and ferroptosis remains to be clarified.

Herein, we demonstrated that CagA promoted ether lipid biosynthesis to increase ferroptosis susceptibility through AGPS and AGPAT3. We further found that this process was mediated through the MEK/ERK/SRF axis. Activation of the MEK/ERK/SRF axis upregulated the expression of the PUFA-ePL biosynthetic enzymes AGPS and AGPAT3 and induced a ferroptosis-susceptible state in GC cells. Moreover, we found that CagA-positive cells were more sensitive to apatinib than were CagA-negative cells. Our findings contribute to developing precise treatment strategies for gastric cancer patients infected with CagA+ *H. pylori* strains.

## Materials and methods

### Cell lines and cell culture

The GC cell lines AGS (RRID:CVCL_0139) and SNU-216 (RRID:CVCL_3946) were obtained from the Type Culture Collection of the Chinese Academy of Sciences (Shanghai, China). All GC cells were cultured in Dulbecco’s modified Eagle’s medium (DMEM, Gibco) supplemented with 10% FBS (Biological Industries) in a 37 °C humidified incubator with a 5% CO_2_ atmosphere.

### Preparation of CagA-expressing GC cells

Transduction of GC cells with the CagA (derived from *H. pylori* strain 26695) expression vector pReceiver-Lv245-CagA with a myc tag or the control empty vector was carried out using a Lenti-Pac™ HIV Expression Packaging Kit, and successfully transduced cell clones were selected using puromycin. The levels of CagA expression in the stable transductants were examined by western blot analysis using whole-cell lysates.

### H. pylori culture and coculture with GC cells

*H. pylori strain 26695 (cagPAI*+*; ATCC 700392)* was cultured on blood agar plates containing 5% sheep serum at 37 °C in a microaerobic atmosphere using the Campy Container System. The mutant strains were derived from *H. pylori strain 26695*. When the GC cells reached 70% confluence, they were serum-starved overnight before *H. pylori* was added at a multiplicity of infection of 100.

### Gastric specimens and H. pylori CagA genotyping

A total of 42 human gastric tumor and corresponding normal gastric tissues were obtained from surgical samples from patients without adjuvant therapy. The clinical characteristics of all patients are listed in Supplementary Table 1, and all specimens were provided by Nanfang Hospital. The study protocol was approved by the Ethics Committee of Nanfang Hospital. Tissue and bacterial DNA were extracted using the TIANamp Bacteria DNA Kit. To confirm the presence of *H. pylori* DNA and the CagA status, 16S rRNA, urea, and CagA were amplified from genomic DNA isolated from bacterial and tissue samples by polymerase chain reaction (PCR)^20,21^. The primers used are listed in Supplementary Table 2.

### Immunohistochemistry

Immunohistochemical staining was performed using 5-μm-thick sections of 4% paraformaldehyde-fixed paraffin-embedded tissue samples. The following primary antibodies were used: anti-AGPAT3 antibody (1:200; DF3642; Affinity; RRID: AB_2836014), anti-4HNE monoclonal antibody (5 µg/ml; Jaica), and anti-AGPS antibody (1:700; ab236621; Abcam; RRID: AB_2921211). Goat anti-rabbit IgG and goat anti-mouse IgG were used as secondary antibodies. Immunohistochemical staining was performed using a Vector DAB kit. Notably, immunohistochemical staining was performed to examine the expression profiles of AGPS and AGPAT3 in gastric specimens as described previously; the expression profiles were evaluated and scored by two pathologists at Nanfang Hospital for the staining intensity (scale = 0–3) and staining frequency (scale = 0–4). For statistical analysis, the expression levels of the AGPS and AGPAT3 proteins were represented by an expression score ranging from 0 to 12 and calculated using the formula intensity×frequency.

### Cell viability assays

For cell viability assays of patient-derived cancer cells, cells were seeded in 94-well opaque white tissue culture plates (Corning) at 5000 cells/well. Eighteen to twenty-four hours after seeding, the cells were treated with the indicated concentrations of the test compounds for the indicated times. The cellular ATP level was quantified using a CellTiter-Glo 2.0 Assay. The relative viability was determined by normalization to that under the corresponding DMSO-treated condition unless otherwise indicated.

The viability of GC cell lines was measured with a Cell Counting Kit-8. Cells were seeded into 96-well plates at 5000 cells/well and cultured for 18–24 h. GC cells were treated with compounds at the indicated concentrations for the indicated times. For the CCK-8 assay, 10 μl of CCK-8 solution was added to each well, and the cells were incubated at 37 °C for 2 h. Then, the absorbance of each well at 450 nm (OD450) was measured using a microplate reader. The relative viability was determined by normalization to that under the corresponding DMSO-treated condition unless otherwise indicated. Three independent experiments were performed.

### Western blot analysis

Cultured cells and GC tissues were lysed in cell lysis buffer supplemented with a protease inhibitor cocktail and phosphatase inhibitor cocktail. The protein concentration was measured using a BCA protein assay. The following primary antibodies were used: anti-4HNE monoclonal antibody (1 µg/ml; MHN-020P; Jaica), anti-SRF polyclonal antibody (1:1000; 16821-1-AP; Proteintech; RRID:AB_2194384), anti-AGPS antibody (1:1000; ab236621; Abcam; RRID:AB_2921211), anti-phospho-ERK1/2 (Thr202/Tyr204) polyclonal antibody (1:1000; 28733-1-AP; Proteintech; RRID:AB_2881202), anti-ERK1/2 polyclonal antibody (1:1000; 51068-1-AP; Proteintech; RRID:AB_2250380), anti-phospho-MEK1 (Ser298) monoclonal antibody (1:1000; 68047-1-Ig; Proteintech; RRID:AB_2918789), anti-MEK1/2 polyclonal antibody (1:1000; 11049-1-AP; Proteintech; RRID:AB_2140649), and anti-β-actin monoclonal antibody (1:1000; 66009-1-Ig; Proteintech; RRID: AB_2687938), and anti-AGPAT3 polyclonal antibody (1:1000; 25723-1-AP; Proteintech; RRID:AB_2880209). The following secondary antibodies were used: HRP-conjugated Affinipure Goat Anti-Rabbit IgG(H + L) (1:5000; SA00001-2; Proteintech; RRID: AB_2722564) and HRP-conjugated Affinipure Goat Anti-Mouse IgG(H + L) (1:5000; SA00001-1; Proteintech; RRID: AB_2722565).

### RNA extraction and quantitative RT‒PCR

Total RNA was extracted using an RNA-Quick Purification Kit, and cDNA was synthesized using HiScript® II QRT SuperMix for qPCR (R222-01; Vazyme). The mRNA transcripts were amplified using the primers listed in Supplementary Table 3, and the relative expression level of each target gene was determined by normalization to the corresponding β-actin mRNA level.

### RNAi-mediated gene knockdown

siRNAs were obtained from Tsingke Biotechnology (Beijing, China). The sequences are listed in Supplementary Table 4. GC cells were transfected with the specified siRNA or scrambled negative control (si NC) siRNA using Lipofectamine 3000 and incubated for 72 h prior to further experiments.

### Patient-derived cancer cell model

GC specimens were placed in sterile conical tubes containing DMEM supplemented with 10% FBS, 1% penicillin–streptomycin, and 1× Primocin within 10 min after tissue separation on wet ice. The tissue digestion mixture was placed in 15-mL centrifuge tubes with 5 mL of DMEM, 1% penicillin–streptomycin, 0.2 mg/mL hyaluronidase and 0.3 mg/mL collagenase. The tubes were placed on a rotator and incubated at 37 °C for 1 h. The cells were then centrifuged at 1000 rpm for 3 min. The cell pellets were resuspended in DMEM supplemented with 10% FBS. Then, the suspended cells were plated into a 96-well plate. The clinical information of the GC patients is provided in Supplementary Table 5.

### Luciferase reporter assay

The luciferase reporter assay was performed in accordance with the manufacturer’s instructions (Luc-Pair™ Duo-Luciferase HS Assay Kit). AGS cells grown in 6-well plates were transiently cotransfected with the reporter plasmid pEZX-PL01-AGPS (2000 ng/well), which contains the 5’-UTR (from −2000 bp to +100 bp) of the human AGPS gene, and the pcDNA3.1-SRF plasmid or control empty vector plasmid (both 1,500 ng/well) in accordance with the manufacturer’s instructions (Lipofectamine 3000; Invitrogen).

### Metabolomic profiling

Sample extracts were analyzed using an LC‒ESI‒MS/MS system at Wuhan Metware Biotechnology Co., Ltd. (Wuhan, China). The cell samples were removed from the −80 °C freezer and placed on ice. Then, 1 mL of extraction solvent (MTBE:MeOH = 3:1, v/v) containing the internal standard mixture was added. After the mixture was whirled for 15 min, 200 μl of water was added, and the mixture was vortexed for 1 min and centrifuged at 12,000 rpm for 10 min. Afterward, 200 μl of the upper organic layer was collected and evaporated using a vacuum concentrator. The dry extract was reconstituted using 200 μl of mobile phase B prior to LC–MS/MS analysis.

The analytical conditions for UPLC were as follows: column, Thermo Accucore™ C30 (2.6 μm, 2.1 mm*100 mm i.d.); solvent system, A: acetonitrile/water (60/40, v/v; 0.1% formic acid; 10 mmol/L ammonium formate); B: acetonitrile/isopropanol (10/90 v/v; 0.1% formic acid; 10 mmol/L ammonium formate); gradient program, A:B (80:20, v/v) at 0 min, 70:30 v/v at 2.0 min, 40:60 v/v at 4 min, 15:85 v/v at 9 min, 10:90 v/v at 14 min, 5:95 v/v at 15.5 min, 5:95 v/v at 17.3 min, 80:20 v/v at 17.3 min, 80:20 v/v at 20 min; flow rate, 0.35 mL/min; temperature, 45 °C; injection volume, 2 μl. LIT and triple quadrupole (QQQ) scans were acquired on a triple quadrupole-linear ion trap mass spectrometer (QTRAP) LC-MS/MS System equipped with an ESI Turbo Ion-Spray interface operating in positive and negative ion mode and controlled by Analyst 1.6.3 software (Sciex).

### Lipid peroxidation assay

For imaging, GC cells were seeded in confocal dishes and then incubated with DMSO, RSL3, erastin, or apatinib for 12 h. During the last 20 min of incubation, 5 μM BODIPY-581/591 C11 was added to the culture medium for live-cell imaging. The cells were imaged at 40× magnification using a Zeiss LSM980 microscope equipped with 405, 488, 543, 594, and 639 nm lasers. All the images were collected with the same instrument parameters and processed with the same settings to maximize the ability to compare the results between conditions. For flow cytometric analysis, GC cells were treated with DMSO, RSL3, erastin, or apatinib for 12 h. The cells were washed with PBS and then incubated with 5 μM BODIPY-C11 for 20 min. Before flow cytometry, the cells were washed with PBS twice, trypsinized, filtered into single-cell suspensions and transferred to FACS tubes. The MDA concentration was measured using an MDA Assay Kit according to the manufacturer’s instructions. The protein concentration was measured using a BCA protein assay kit.

### Chromatin immunoprecipitation (ChIP) assay

ChIP was carried out using the SimpleChIP Enzymatic Chromatin IP Kit (Magnetic Beads) according to the manufacturer’s protocol. The cells were subjected to crosslinking in 1% formaldehyde for 10 min, and glycine was then added for 5 min. After washing with cold PBS, the cells were collected and incubated with micrococcal nuclease for 20 min at 37 °C, after which the nuclear membrane was lysed by 3 sets of 20-s pulses using an ultrasonicator. Then, 50 μl of each sonicated sample was removed to determine the DNA concentration and fragment size. The cell lysates were incubated overnight with 2 µg of normal rabbit IgG as the negative control, 2 µg of a ChIP-grade anti-SRF antibody (Active Motif), or 10 µl of an anti-Histone H3 (D2B12) XP rabbit mAb at 4 °C. Then, 30 µl of Protein G magnetic beads was added to each IP reaction, and the mixtures were incubated for 2 h at 4 °C with rotation. The beads were collected, washed, and treated with proteinase K for 2 h at 65 °C. DNA was purified on spin columns, and the DNA fragments were analyzed via qRT–PCR. All DNA levels were normalized to those in the input DNA sample.

### Animal experiments

For the xenograft models, male nu/nu mice aged 5–6 weeks were used. The mice were purchased from Guangdong Medical Laboratory Animal Center. All procedures involving animals and their care in this study were performed in accordance with guidelines approved by the Southern Medical University Institutional Animal Care and Use Committee. The athymic nude mice were injected subcutaneously with five million SNU-216-CagA cells or control cells. The mice were randomized into groups. For (1S, 3R)-RSL3 treatment, mouse xenografts were allowed to grow for 1 week prior to intratumoral injection of vehicle (PEG-400) or 50 mg/kg (1S, 3R)-RSL3 twice per week for 2 weeks^22^. The tumor size was measured 4 weeks after cell injection. For apatinib treatment, mouse xenografts were allowed to grow for 1 week, and 50 mg/kg apatinib was administered to the mice orally every day for 3 weeks. For liproxstatin-1 treatment, 20 mg/kg liproxstatin-1 was administered daily via the i.p. route. Tumor volumes were measured by measuring the length (L) and width (W) of each tumor using a caliper at 3-day intervals. Tumor volumes were calculated with the following equation: V = (L*W*W)/2.

### Statistical analysis

The data are presented as the mean ± SD unless stated otherwise. All the statistical analyses were performed by using Prism 9 (GraphPad Software). The significance of differences was evaluated using 2-tailed Student’s *t*-test or 1-way ANOVA. A *P* value of less than 0.05 was considered to indicate statistical significance.

## Results

### CagA confers GC cells sensitivity to ferroptosis

**Fig. 1:**
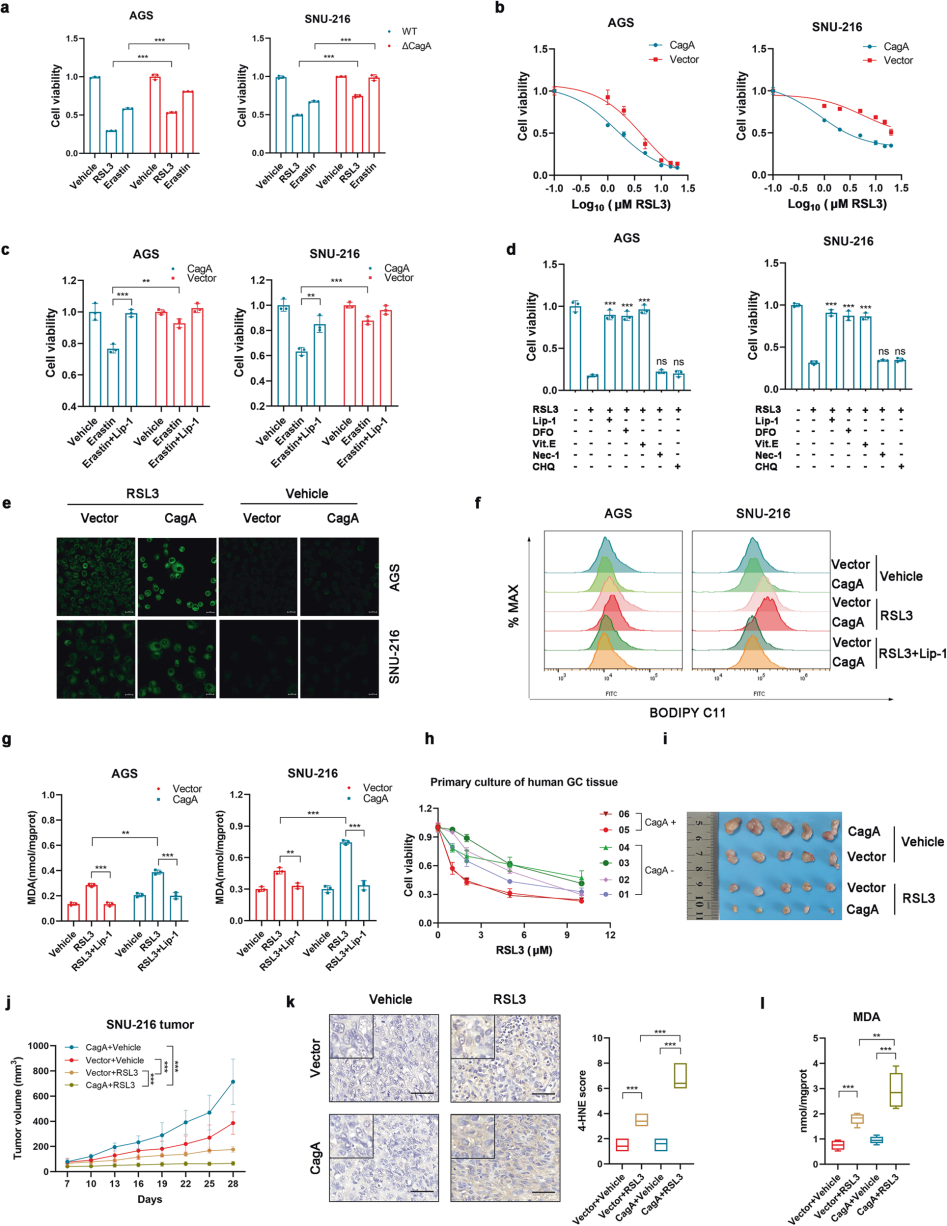
CagA promotes ferroptosis in GC cells. **a** Gastric cancer cells were infected with *H. pylori 26695* or the isogenic CagA deletion mutant strain for 24 h and then treated with RSL3 (10 µM for AGS and 15 µM for SNU-216) or erastin (15 µM for AGS and 20 µM for SNU-216) for 24 h. **b** Relative viability of the indicated GC cells treated with RSL3 for 24 h. **c** Relative viability of the indicated GC cells treated with erastin (20 µM), liproxstatin-1 (Lip-1, 2 µM), or both for 24 h. **d** Relative viability of AGS-CagA and SNU-216-CagA cells treated with RSL3 (10 µM for AGS and 15 µM for SNU-216) plus the indicated concentrations of DMSO, Lip-1 (2 µM), deferoxamine (DFO, 100 µM), vitamin E (Vit. E, 10 μM), chloroquine (CHQ, 50 µM), or necrostatin-1 (Nec-1, 25 µM) for 24 h. **e** Fluorescence images of BODIPY-C11-stained GC cells treated with DMSO or RSL3 (5 µM for AGS and 10 µM for SNU-216) for 12 h. Scale bars: 20 µm. **f** Flow cytometric analysis of the indicated BODIPY-C11-stained GC cells treated with DMSO or RSL3 (5 µM for AGS and 10 µM for SNU-216) and/or liproxstatin-1 (100 nM) for 12 h. **g** Changes in cellular malondialdehyde levels in the indicated GC cell lines treated with DMSO or RSL3 and/or liproxstatin-1 (100 nM) for 12 h. **h** Relative viability of patient-derived primary GC cell lines treated with RSL3 for 16 h. **i** Gross images of tumor tissues from nude mice after drug treatments (*n* = 5). **j** Tumor growth curves. (**k**) Paraffin-embedded tumor sections stained for 4HNE. Representative images are shown; scale bar = 50 µm. The IHC signals were scored. (**l**) Changes in cellular malondialdehyde levels in tumor tissues from nude mice after drug treatments. The data are presented as the mean ± SD; ns, nonsignificant; *p < 0.05; **p < 0.01; ***p < 0.001.

To test whether CagA is linked to ferroptosis, we infected gastric cancer cells with the bacterial strain *26695* (CagA-positive *H. pylori*) or the isogenic CagA deletion mutant strain. Ferroptosis was induced by treatment with a class 1 ferroptosis inducer (erastin, which blocks SLC7A11-mediated cystine transport) or a class 2 ferroptosis inducer (RSL3, which inhibits GPX4 activity)^11,22^. Compared with cells infected with the isogenic CagA deletion mutant strain, cells infected with the WT strain exhibited increased sensitivity to ferroptosis inducers (Fig. 1a). Consistently, AGS and SNU-216 cells stably expressing CagA were also susceptible to RSL3-induced and erastin-induced ferroptosis (Fig. 1b, c, Supplementary Fig. 1a). The CagA-induced sensitivity to RSL3 and erastin could be rescued by treating cells with the ferroptosis inhibitors liproxstatin-1 (Lip-1), deferoxamine (DFO), and vitamin E (Vit. E) but not by treating cells with the autophagy inhibitor chloroquine (CHQ) or the RIPK1 inhibitor necrostatin-1 (Nec-1) (Fig. 1c, d). Ferroptosis is a lipid peroxidation-driven form of regulated cell death (RCD)^23,24^. Therefore, we determined the level of lipid peroxidation by imaging and flow cytometry using the lipid peroxidation sensor BODIPY-C11 and found that CagA expression significantly increased lipid peroxidation (Fig. 1e, f). Moreover, the levels of the end product of lipid peroxidation (MDA) and the ferroptosis marker *PTGS2* were significantly higher in CagA-expressing GC cells than in control cells following treatment with RSL3 (Fig. 1g, Supplementary Fig. 1b). Liproxstatin-1 reduced lipid peroxidation induced by RSL3 in both CagA-expressing GC cells and control cells (Fig. 1f, g). This susceptibility to ferroptosis was recapitulated in the colony formation assay (Supplementary Fig. 1c), in patient-derived primary GC cell lines (Fig. 1h) and in SNU-216-CagA xenografts in vivo (Fig. 1i, j, and Supplementary Fig. 1d). Consistently, in xenograft tumors treated with RSL3, the levels of 4-hydroxynonenal (4-HNE, a marker of oxidative stress-induced lipid peroxidation and cytotoxicity), MDA, and *PTGS2* mRNA were generally higher in the CagA group than in the control group (Fig. 1k, l, and Supplementary Fig. 1e). Collectively, these results show that CagA confers cell sensitivity to ferroptosis, revealing a therapeutic approach for inducing ferroptosis as a treatment strategy for GC patients infected with CagA+ *H. pylori* strain.

### AGPS and AGPAT3 mediate sensitivity to ferroptosis in CagA-expressing GC cells

**Fig. 2:**
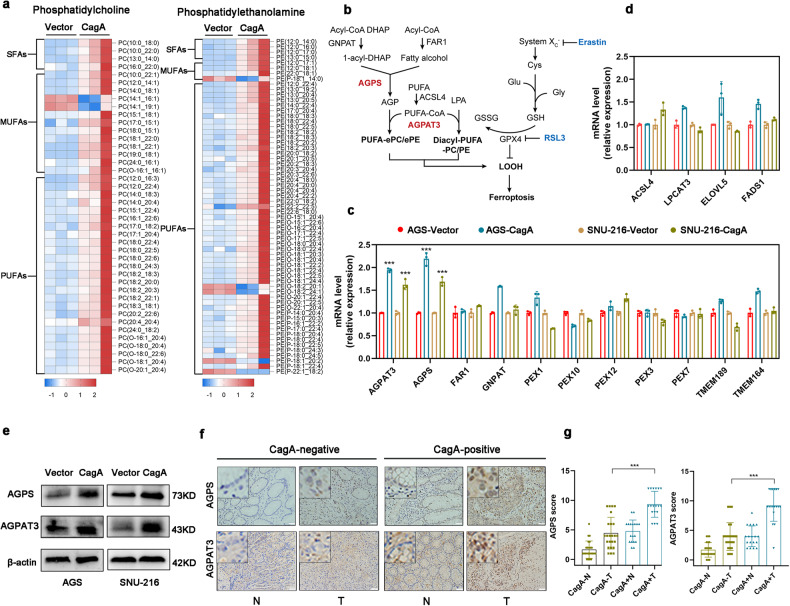
AGPS- and AGPAT3-mediated proferroptotic effects of CagA. **a** The differential expression of PC and PE species was analyzed by mass spectrometry in SNU-216-CagA cells (*n* = 3 samples) and SNU-216-Vector cells (*n* = 3 samples). (PE-O, phosphatidylethanolamine ether phospholipids; PC-O, phosphatidylcholine ether phospholipids; PE-P, phosphatidylethanolamine vinyl-ether phospholipids). The lipid content was normalized to the infused protein for each condition and replicate. **b** PUFA-ePLs and PUFA-PLs acted as substrates for lipid peroxidation, which induces ferroptotic cell death. (**c**, **d**) Fold changes in the expression of genes encoding enzymes related to ePL biosynthesis, polyunsaturated fatty acid synthesis, or fatty acid elongation in CagA-expressing GC cells compared with empty vector-containing cells. **e** Western blots showing the protein levels of AGPS and AGPAT3 in the indicated GC cells. **f** Representative images of AGPS and AGPAT3 immunoreactivity (brown) in gastric cancer tissues (T) and the corresponding normal gastric tissues (N) from CagA-positive (*n* = 18) and CagA-negative GC patients (*n* = 24), respectively. Scale bar = 50 µm. **g** The IHC signals were scored to evaluate AGPS and AGPAT3 protein expression. The data are presented as the mean ± SD; ns, nonsignificant; *p < 0.05; **p < 0.01; ***p < 0.001.

As the cellular lipid composition and fatty acid metabolism are known to control ferroptosis susceptibility in cells^12,13,25–27^, we next sought to investigate whether CagA induces a ferroptosis-susceptible state through lipid modulation in GC cells. We conducted lipid profiling using liquid chromatography–tandem mass spectrometry (LC‒MS/MS) on SNU-216-CagA cells and empty vector-containing cells. Lipidomic analysis revealed 1614 different lipid species, consisting of phosphatidylcholines (PCs), triglycerides (TGs), phosphatidylethanolamines (PEs), and other lipid classes (Supplementary Table 6). The levels of PEs and PCs were generally increased in CagA-expressing cells, whereas the TGs levels were decreased in these cells. In addition to its effect on diacyl PE and diacyl PC species, CagA induced the accumulation of ether lipids, such as PE plasmalogen (PE-P), PE-O, and PC-O species (Fig. 2a). PUFA-ePLs and PUFA-PLs acted as substrates for lipid peroxidation, which induces ferroptotic cell death^13^ (Fig. 2b). In contrast, MUFAs can limit lipid peroxidation and ferroptosis^28^. We found that most diacyl PE and diacyl PC species with different double bonds were upregulated in SNU-216-CagA cells. Notably, the levels of most detected polyunsaturated ether phospholipids (PUFA-ePLs) with three or more double bonds were found to be high in SNU-216-CagA cells. We next measured the mRNA levels of the key enzymes involved in ePL- biosynthesis, polyunsaturated fatty acid synthesis, or fatty acid elongation, including alkylglycerone phosphate synthase (*AGPS*), 1-acylglycerol-3-phosphate O-acyltransferase 3 (*AGPAT3*), fatty acyl-CoA reductase 1 (*FAR1*), peroxisomal biogenesis factor 1 (*PEX1*), peroxisomal biogenesis factor 3 (*PEX3*), peroxisomal biogenesis factor 7 (*PEX7*), peroxisomal biogenesis factor 10 (*PEX10*), peroxisomal biogenesis factor 12 (*PEX12*), and glycerone-phosphate O-acyltransferase (*GNPAT*), plasmanylethanolamine desaturase 1 (*TMEM189*); transmembrane protein 164 (*TMEM164)*, acyl-CoA synthetase long-chain family member 4 (*ACSL4*), lysophosphatidylcholine acyltransferase 3 (*LPCAT3*), elongation of very long-chain fatty acid protein 5 (*ELOVL5*), and fatty acid desaturase 1 (*FADS1*)^29,30^. We found that *AGPS* and *AGPAT3* were expressed at higher levels in CagA-expressing GC cells than in empty vector-containing cells (Fig. 2c, d). Consistently, CagA upregulation increased the protein levels of AGPS and AGPAT3 in CagA-expressing GC cells (Fig. 2e). We next examined AGPS and AGPAT3 expression in human gastric biopsy specimens by immunohistochemistry and western blotting. Consistent with our cell culture data, the expression levels of AGPS and AGPAT3 were increased in gastric epithelial cells harvested from GC patients infected with CagA+ *H. pylori* compared with those in GC patients infected with CagA- *H. pylori* or not infected with *H. pylori* (Fig. 2f, g, and Supplementary Fig. 2a). It was reported that AGPAT3 depletion specifically reduced the number of ePLs containing PUFAs^13^. The expression of AGPS and AGPAT3 might play a key role in ferroptosis susceptibility mediated by CagA.

### Inhibition of the expression of AGPS and AGPAT3 blocks ferroptosis susceptibility mediated by CagA

**Fig. 3:**
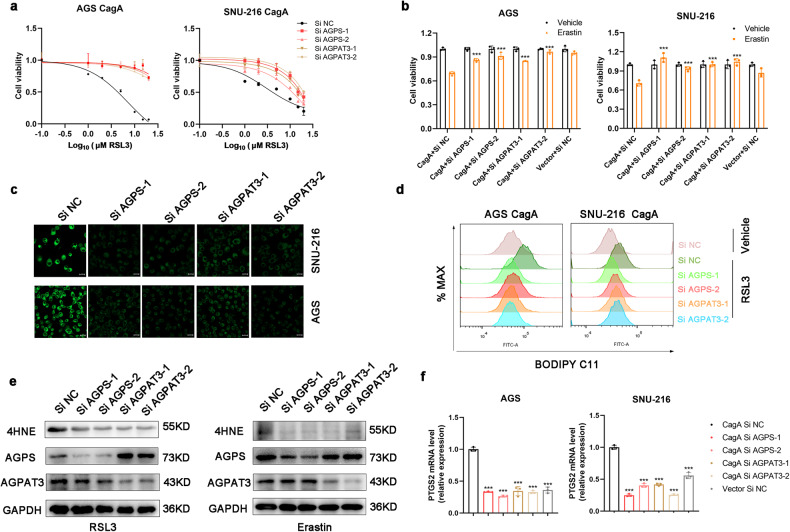
Inhibition of AGPS and AGPAT3 expression blocks ferroptosis susceptibility mediated by CagA. **a**, **b** Relative viability of WT, AGPS-knockdown, and AGPAT3-knockdown GC cells treated with RSL3 or erastin. **c,** **d** Fluorescence images and flow cytometric analysis of BODIPY-C11-stained WT, AGPS-knockdown, and AGPAT3-knockdown GC cells treated with DMSO or RSL3 for 12 h. Scale bars: 20 µm. **e** The 4HNE protein level was measured by western blotting in WT, AGPS-knockdown, and AGPAT3-knockdown AGS cells treated with RSL3 or erastin. **f** PTGS2 mRNA level in WT, AGPS-knockdown, and AGPAT3-knockdown GC cells treated with RSL3. The data are presented as the mean ± SD; *p < 0.05, **p < 0.01, ***p < 0.001.

To ascertain whether AGPS and AGPAT3 indeed play key roles in CagA-mediated ferroptosis susceptibility, we assessed the responses of cells with knockdown of AGPS or AGPAT3. The results showed that siRNA-mediated AGPS and AGPAT3 knockdown prevented RSL3-induced and erastin-induced cell death in AGS-CagA and SNU-216-CagA cells (Fig. 3a, b). We also found that RSL3 effectively abrogated the clonogenic survival of SNU-216-CagA cells, which was restored by knockdown of AGPS or AGPAT3 (Supplementary Fig. 2b). We next measured the levels of lipid peroxidation following treatment with RSL3 and found that, compared with control cells, cells with knockdown of AGPS or AGPAT3 exhibited decreases in lipid peroxidation, the 4HNE protein level, and the *PTGS2* mRNA level (Fig. 3c–f). Overall, these data showed that the ether lipid biosynthesis enzymes AGPS and AGPAT3 play essential roles in sensitivity to CagA-mediated ferroptosis.

### The CagA-mediated increases in AGPS expression, AGPAT3 expression and ferroptosis susceptibility are dependent on the MEK/ERK pathway

**Fig. 4:**
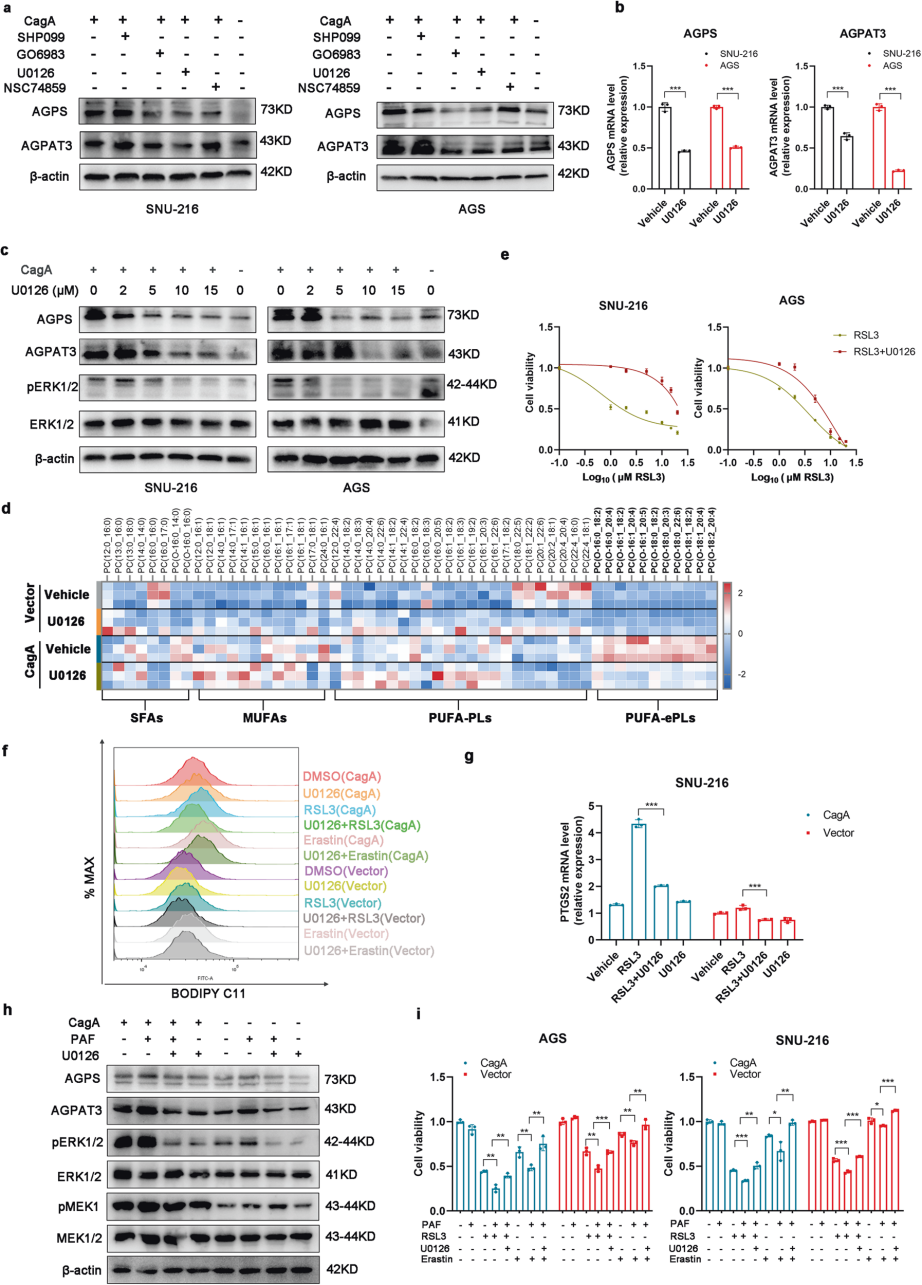
CagA-induced upregulation of AGPS and AGPAT3 is mediated by activation of the MEK/ERK pathway. **a** Western blot analysis of AGPS and AGPAT3 expression in GC cells treated with chemical inhibitors (at a final concentration of 10 μM) of the indicated enzymes for 24 h. **b** qRT‒PCR analysis of AGPS and AGPAT3 expression in GC cells treated with U0126 (10 μM) for 24 h. **c** Western blot analysis of AGPS and AGPAT3 protein expression in GC cells treated with various concentrations of U0126 for 24 h. **d** Heatmap showing the relative amounts of PC species in SNU-216-CagA cells treated with U0126 (10 µM) for 24 h. Highlighted in bold are PUFA-containing ether lipids. **e** Relative viability of the indicated GC cells treated with RSL3 alone or in combination with U0126 (10 µM) for 24 h. **f** Lipid peroxidation was evaluated by BODIPY 581/591 C11 staining of AGS cells treated with RSL3 (5 µM) or erastin (10 µM) alone or in combination with U0126 (10 µM) for 12 h. **g** Fold changes in the expression of PTGS2 in GC cells treated with RSL3 (10 µM), U0126 (10 µM), or both for 12 h. **h** Western blot analysis of AGPS and AGPAT3 protein expression in GC cells treated with PAF (10 µM), U0126 (10 µM), or both for 24 h. **i** Viability of GC cells treated with RSL3 (5 µM for AGS, 10 µM for SNU-216) or erastin (15 µM for AGS, 20 µM for SNU-216) alone or in combination with PAF (10 µM) and U0126 (10 µM) for 24 h. The data are presented as the mean ± SD; *p < 0.05, **p < 0.01, ***p < 0.001.

To explore how the expression of AGPS and AGPAT3 is affected by CagA, we performed a series of experiments using a panel of chemical inhibitors of various proteins that were previously reported to be activated by CagA, including MEK1/2, PKC, SHP2, and STAT3^31–34^. Among the tested compounds, we found that inhibitors of MEK1/2 downregulated the mRNA and protein expression of AGPS and AGPAT3 in a dose-dependent manner (Fig. 4a–c). Lipidomic analysis also revealed that the MEK1/2 inhibitor U0126 selectively reduced the levels of largely polyunsaturated ether phospholipids (PUFA-ePLs) in SNU-216-CagA cells (Fig. 4d, Supplementary Fig. 3a, Supplementary Table 7). In addition, U0126 restored cell viability, prevented lipid peroxidation, and decreased the *PTGS2* mRNA level in AGS-CagA and SNU-216-CagA cells treated with RSL3 or erastin (Fig. 4e–g). Immunoblot analysis showed that U0126 decreased the 4HNE level in SNU-216-CagA cells treated with RSL3 (Supplementary Fig. 3b). To confirm these findings and minimize off-target effects, we treated GC cells with the more selective and potent MEK1/2 inhibitor PD0325901 and found that PD0325901 downregulated the protein expression of AGPS and AGPAT3 in a dose-dependent manner (Supplementary Fig. 3c). PD0325901 was able to block the induction of ferroptosis susceptibility in CagA-expressing GC cells by RSL3 (Supplementary Fig. 3d, e). Conversely, the MEK/ERK activator C16-PAF increased the expression of AGPS and AGPAT3 (Fig. 4h) and induced sensitivity to ferroptosis in AGS and SNU-216 cells, but these effects were blocked by cotreatment with U0126 (Fig. 4i). Thus, MEK/ERK pathway activation could upregulate the expression of the AGPS and AGPAT3 proteins.

### MEK/ERK-induced upregulation of AGPS and AGPAT3 in CagA-expressing GC cells is mediated by SRF

**Fig. 5:**
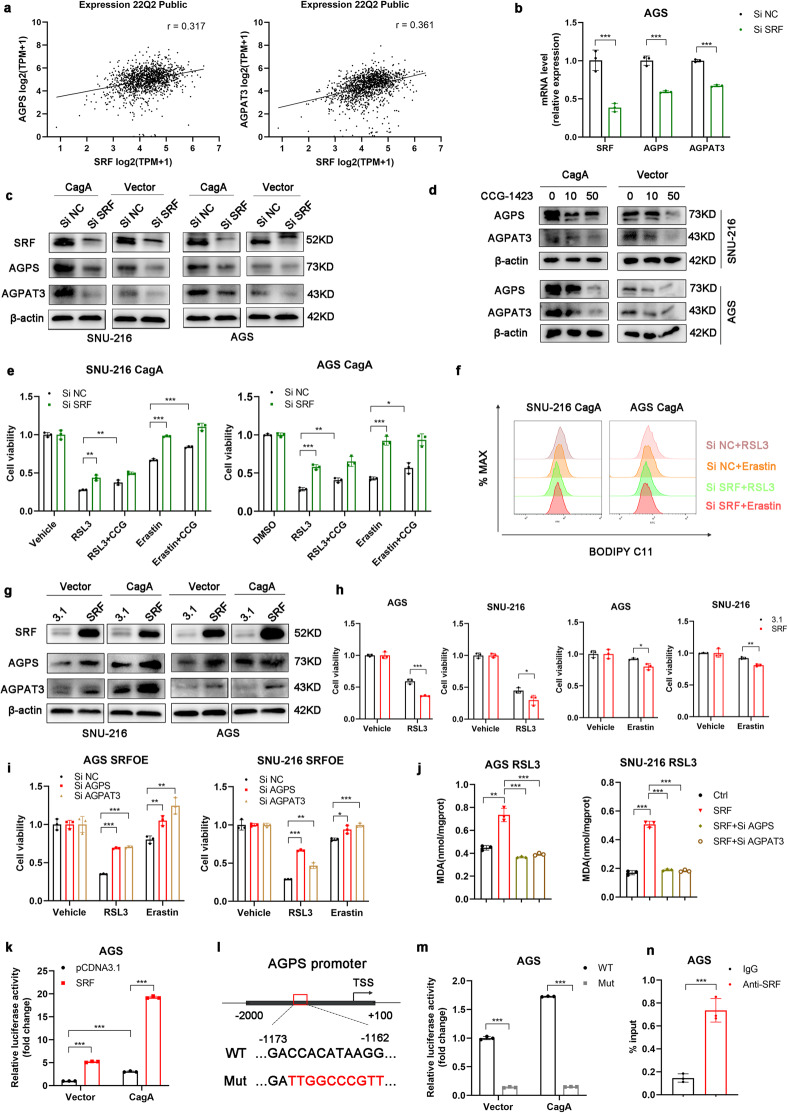
CagA-induced expression of AGPS was mediated by the transcriptional activator SRF. **a** Analysis of the correlation between SRF and AGPS or AGPAT3 in cell lines in a public database [https://depmap.org/portal/]. Significance was evaluated by Pearson correlation analysis. **b**, **c** AGPS and AGPAT3 expression after siRNA transfection was assessed by qRT‒PCR and western blotting. **d** AGPS and AGPAT3 protein levels were measured by western blotting in the indicated GC cells treated with vehicle or CCG-1423 (10 μM or 50 μM). **e** Relative viability of the indicated GC cells treated with RSL3 (5 µM for AGS, 15 µM for SNU-216) or erastin (20 µM) alone or in combination with CCG-1423 (10 μM) for 24 h. **f** Cellular lipid peroxidation was evaluated by BODIPY 581/591 C11 staining using flow cytometry. **g** The protein levels of AGPS and AGPAT3 in GC cells transfected with pcDNA3.1 or pcDNA3.1-SRF. **h** Relative viability of SRF-overexpressing GC cells compared to empty vector control cells treated with RSL3 (5 µM for AGS, 15 µM for SNU-216) or erastin (20 µM) for 24 h. **i,** **j** Treatment with RSL3 (5 µM for AGS, 15 µM for SNU-216) or erastin (20 µM) for 72 h in SRF-overexpressing GC cells with siRNA-mediated knockdown of AGPS and AGPAT3 compared to SRF-overexpressing GC cells with siNC treatment. Cell viability and cellular malondialdehyde levels were measured. **k** Cells cotransfected with the SRF and WT (−2000~+100) plasmid were collected, and luciferase activity was analyzed. **l** Sequences of the wild-type and mutant AGPS sequences linked to the reporters. **m** Cells transfected with the mutant plasmids were subjected to a luciferase assay, and the results were normalized to those of the WT group. **n** Chromatin immunoprecipitation using specific antibodies was used to evaluate the kinetics of the binding of SRF to the AGPS promoter. The data are presented as the mean ± SD; *p < 0.05, **p < 0.01, ***p < 0.001.

Activation of the MEK/ERK signaling pathway regulates serum responsive factor (SRF)-dependent gene expression^35^. According to database (JASPAR) prediction, the AGPS promoter region harbors a binding site for SRF. Moreover, analysis of a public database (DepMap) revealed the coexpression of SRF and AGPS (Fig. 5a). To test whether SRF regulates AGPS expression via its activity as a transcription factor, SRF was downregulated by siRNA in GC cells, which led to significant decreases in the mRNA and protein levels of AGPS (Fig. 5b, c). Interestingly, analysis of a public database also revealed the coexpression of SRF and AGPAT3 (Fig. 5a), and SRF siRNA decreased the CagA-induced expression of AGPAT3 (Fig. 5b, c). Similar to siRNA, CCG-1423-mediated inhibition of SRF suppressed the expression of AGPS and AGPAT3 (Fig. 5d and Supplementary Fig. 3f). In addition, inhibition of SRF by siRNA transfection or CCG-1423 treatment in CagA-expressing GC cells attenuated the decrease in cell viability caused by treatment with RSL3 or erastin (Fig. 5e). Furthermore, inhibition of SRF decreased lipid peroxidation following treatment with RSL3 or erastin, as indicated by the reduced levels of green fluorescence (BODIPY 581/591 C11), *PTGS2* mRNA, MDA, and 4HNE (Fig. 5f and Supplementary Fig. 3g-i). In contrast, introduction of pcDNA3.1-SRF increased the mRNA and protein levels of AGPS and AGPAT3 (Fig. 5g). The level of ferroptosis was significantly increased by SRF overexpression in AGS and SNU-216 cells following treatment with RSL3 or erastin (Fig. 5h). However, this effect was blocked by treatment with CCG-1423 (Supplementary Fig. 3j), suggesting that SRF can promote ferroptosis. As expected, SRF overexpression-mediated ferroptosis was markedly inhibited by AGPS and AGPAT3 depletion in GC cells (Fig. 5i, j, and Supplementary Fig. 3k). Collectively, these data demonstrate that SRF overexpression significantly sensitizes GC cells to AGPS- and AGPAT3-dependent ferroptosis.

Since SRF promotes AGPS expression at the transcriptional level, to understand how AGPS is induced by SRF, we examined the AGPS promoter and upstream region. Transcription assays using a transiently transfected reporter construct containing the sequence between −2000 and +100 bp upstream of the AGPS translation start site showed significant induction of AGPS promoter activity in both CagA-expressing and control cells cotransfected with the SRF expression plasmid (Fig. 5k). Then, we performed a database analysis to predict the binding motif(s) in the SRF promoter region to verify the binding site (JASPAR). We identified one motif that was similar to the binding sites of the SRF transcription factor family (Fig. 5l). Mutation of this motif blocked AGPS promoter activity (Fig. 5m). Chromatin immunoprecipitation (ChIP) assays showed that SRF bound to the region of the AGPS promoter containing this motif (Fig. 5n). Taken together, these findings show that the MEK/ERK-induced upregulation of AGPS and AGPAT3 in CagA-expressing GC cells is mediated by SRF and that SRF is a crucial transcription factor that increases AGPS transcription by binding to the AGPS promoter region.

### Apatinib demonstrates broad therapeutic efficacy in CagA-positive gastric cancer

**Fig. 6:**
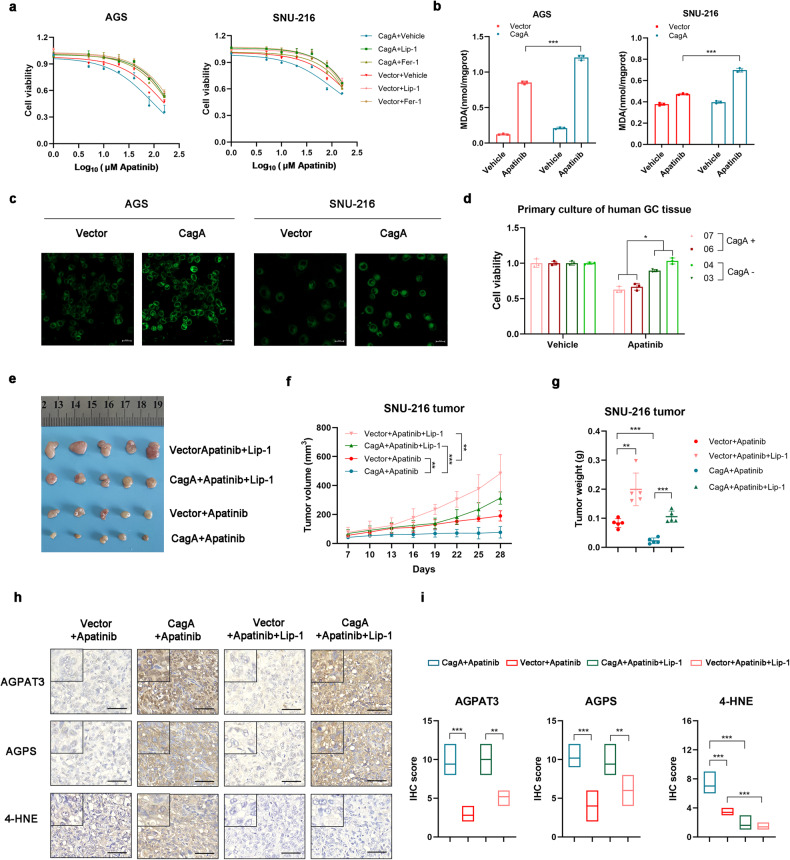
CagA promotes the sensitivity of GC cells to apatinib. **a** Relative viability of the indicated GC cells treated with various concentrations of apatinib, liproxstatin-1 (100 nM), and/or ferrostatin-1 (Fer-1, 1 µM) for 24 h. **b** Changes in cellular malondialdehyde levels in GC cell lines treated with apatinib or vehicle for 12 h. **c** Fluorescence images of BODIPY-C11-stained AGS and SNU-216 cells treated with apatinib for 12 h. Scale bars: 20 µm. **d** Relative viability of patient-derived primary GC cell lines treated with apatinib (50 µM) for 16 h. **e** Gross images of tumor tissues from nude mice after drug treatments (*n* = 5). **f** Tumor growth curves. **g** Tumor weight. **h** Paraffin-embedded tumor sections stained for 4HNE, AGPS, and AGPAT3. Representative images are shown; scale bar = 50 µm. **i** The IHC signals were scored. The data are presented as the mean ± SD; *p < 0.05, **p < 0.01, ***p < 0.001.

We confirmed in our previous study that apatinib induces lipid ROS-dependent ferroptosis in GC cells^36^. To evaluate whether CagA confers sensitivity to apatinib, we measured the viability of GC cells following treatment with apatinib. Remarkably, the results revealed that apatinib sensitivity in CagA-expressing cells was higher than that in control cells and that treatment with liprostatin-1 or ferrostatin-1 partially blocked cell death induced by apatinib both in CagA-expressing cells and control cells (Fig. 6a). Moreover, apatinib treatment induced a greater increase in the level of MDA and more rapid accumulation of lipid radicals in CagA-positive cells than in CagA-negative cells (Fig. 6b, c). We subsequently generated a patient-derived primary GC cell line model. Consistent with our in vitro findings, primary GC cells isolated from patients infected with the CagA+ *H. pylori* strain exhibited substantially higher sensitivity to apatinib than cells isolated from GC patients infected with CagA- *H. pylori* or from uninfected patients (Fig. 6d). Our cell-level studies prompted further analysis of apatinib sensitivity in CagA-expressing cells in vivo. The CagA-expressing group was susceptible to ferroptosis induced by apatinib (Fig. 6e–g), as evidenced by the increase in the 4-HNE staining intensity when residual tumors were stained after the end of treatment, and liproxstatin-1 alleviated this effect (Fig. 6h). CagA expression was observed to increase AGPS and AGPAT3 expression in SNU-216-CagA cell-derived tumors compared with control cell-derived tumors (Fig. 6i). These data provide strong in vivo evidence that detection of the *H. pylori* CagA status may aid patient stratification for treatment with apatinib.

## Discussion

Chronic *H. pylori* infection is considered the principal cause of gastric cancer, making this pathogen the most common infectious agent linked to malignancy^37^. *H. pylori* colonizes the stomach in approximately half of the world’s population^38^. *H. pylori* strains that possess a functional cag PAI are associated with a higher risk for gastric cancer than cag− strains^39^. CagA is delivered into the cytoplasm of gastric epithelial cells during bacterial attachment through the type IV secretion system (T4SS). The global ratio of CagA-positive strains to CagA-negative strains is approximately 6:4, with the notable exception of East Asian countries, where nearly all isolated *H. pylori* strains are CagA-positive^40^. Our study emphasizes the potent activity of CagA in inducing a ferroptosis-susceptible state through lipid modulation in GC cells. Ether lipids make up approximately 20% of the total phospholipid mass in humans^41^. Ether lipids have been reported to play key roles in several pathologies, including neurological disorders, cardiovascular diseases, metabolic disorders, and cancers^14^. Cancer cells are known to have markedly higher levels of ether lipids than normal cells^42,43^. The role of ePLs in the biological behaviors of cancer cells has been studied mainly by modulating the expression of enzymes involved in ePL biosynthesis. CagA does not specifically target gastric cancer cells^44,45^. However, the metabolic state of tumor cells differs from that of normal cells. In this study, AGPS and AGPAT3 were found to be expressed at higher levels in GC tissues, especially in CagA+ *H. pylori*-infected tumor tissues, than in normal tissues. Inactivation of AGPS lowers ether lipid levels and impairs cancer pathogenicity^42^. However, PUFA-ePLs can act as substrates for lipid peroxidation, which induces ferroptotic cell death^13^, and a high-PUFA-ePL state results in dependency on ferroptosis defense pathways to maintain redox homeostasis. By using 1 S,3R-RSL3 (RSL3) and erastin to inhibit the antioxidant response, we found that CagA induced massive lipid peroxidation and sensitized GC cells to ferroptosis in vitro and in vivo.

Our study also highlights the important role of the MEK/ERK/SRF pathway in driving ferroptosis sensitivity and regulating the expression of AGPS and AGPAT3 in cancers. Using a panel of chemical inhibitors of various proteins previously reported to be activated by CagA^32^, we found that MEK1/2 inhibitors downregulated the expression of AGPS and AGPAT3, thereby decreasing sensitivity to ferroptosis in CagA-positive GC cells. CagA-mediated increases in AGPS and AGPAT3 expression and ferroptosis susceptibility are dependent on the MEK/ERK/SRF pathway.

Emerging evidence indicates that the activation of ferroptosis is a promising strategy for eliminating cancer cells that are resistant to drug-induced apoptosis^46–49^. However, due to their poor bioavailability, erastin and RSL3 are not suitable for direct use in vivo^15^. Apatinib, a highly selective inhibitor of the vascular endothelial growth factor receptor-2 (VEGFR2) tyrosine kinase, was approved for the treatment of metastatic gastric cancer by the cFDA^50^. In our previous study, we demonstrated that apatinib induces lipid ROS-dependent ferroptosis in GC cells^36^. In this study, we evaluated the viability of CagA-expressing GC cells and control cells following treatment with apatinib and found that CagA-expressing cells were more sensitive to apatinib than were control cells. We anticipate that detecting the *H. pylori* infection status may aid patient stratification for treatment with apatinib.

In conclusion, our study indicates that CagA-positive GC is sensitive to therapeutic induction of ferroptosis and delineates the genetic and metabolic basis for this susceptibility. The insights gained from this study could inform precise treatment strategies involving ferroptosis for patients with gastric cancer who are infected with CagA+ *H. pylori*.

### Supplementary information

Supplementary materials

Supplementary Table 6

Supplementary Table 7

## Data Availability

The supporting data of this study are available from the corresponding author upon reasonable request.
