# Supplementary material for: Helicobacter pylori CagA-mediated ether lipid biosynthesis promotes ferroptosis susceptibility in gastric cancer
Source: Exp Mol Med. 2024 Feb 21;56(2):441–52. doi: 10.1038/s12276-024-01167-5 (PMC10907675; doi:10.1038/s12276-024-01167-5)
Supplement: Supplementary file 1 — Supplementary materials [file 12276_2024_1167_MOESM1_ESM.pdf]

Supplementary Information

Helicobacter pylori CagA-mediated ether lipid biosynthesis promotes ferroptosis susceptibility in gastric cancer

*Yanmei Peng, Xuetao Lei, Qingbin Yang, Guofang Zhang, Sixiao He, Minghao Wang, Ruoyu Ling, Boyang Zheng, Jiayong He, Xinhua Chen, Fengping Li, Qiming Zhou, Liying Zhao *, Gengtai Ye *, Guoxin Li **

This file includes:

Supplementary Fig. 1 to 3
Supplementary Table 1 to 5

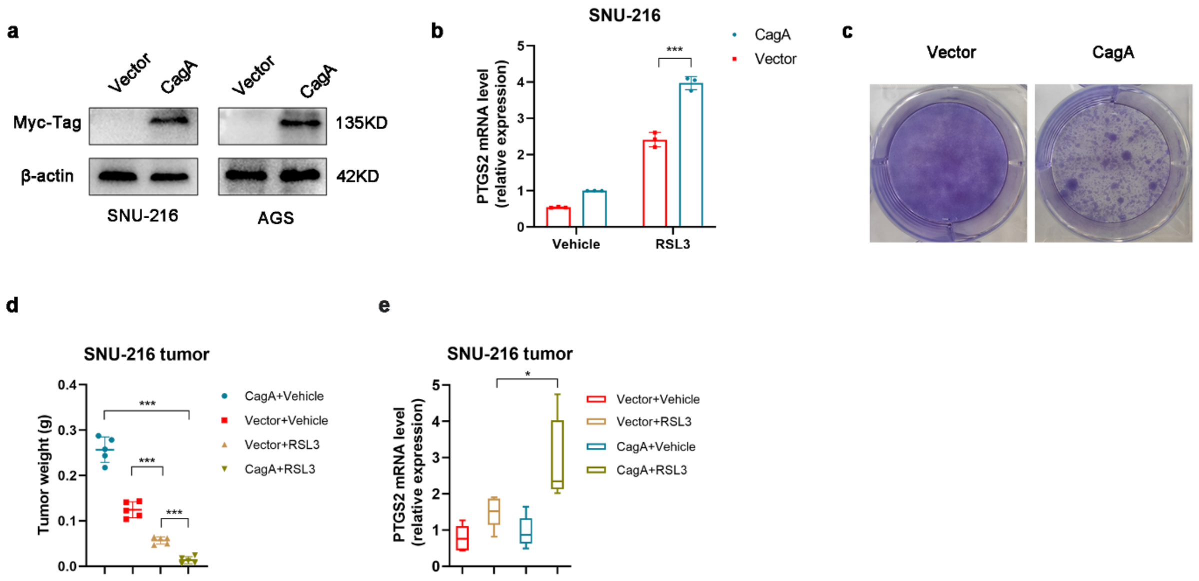

Supplementary Fig. 1. CagA promotes ether lipid biosynthesis through AGPS and AGPAT3 in gastric cancer

(**a**) Myc-tagged CagA expressed in SNU-216 and AGS cells. (**b**) Fold changes in the expression of *PTGS2* in GC cells following treatment with vehicle or RSL3 (10 µM). (**c**) GC cells were treated with RSL3 (5 µM) for 12 h. Cells were washed, cultured for 7 days for recovery and stained with crystal violet. (**d**) Tumor weight. (**e**) Fold changes in the expression of *PTGS2* in tumor tissues from nude mice after drug treatments. Data represented as mean ± SD, *$p < 0.05$, ***$p < 0.001$.

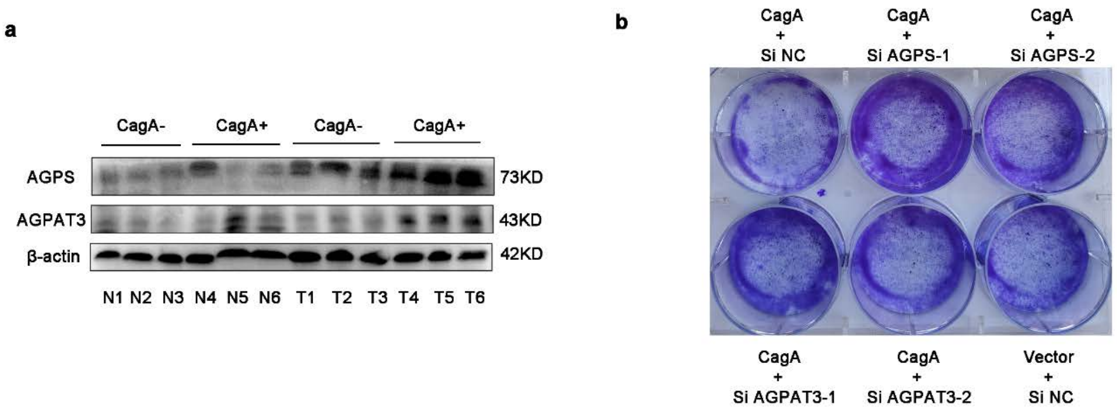

Supplementary Fig. 2. CagA mediates ferroptosis through regulation of AGPS and AGPAT3 in GC cells

(**a**) Representative AGPS and AGPAT3 immunoblot determined in the gastric cancer tissues (T) and their corresponding normal gastric tissues (N) from CagA-postive and CagA-negative GC patients, respectively. (**b**) SNU-216 cells were treated with indicated siRNA for 72h, then treated with RSL3 (5 μM) for 12 h. Cells were washed, cultured for 7 days for recovery and stained with crystal violet.

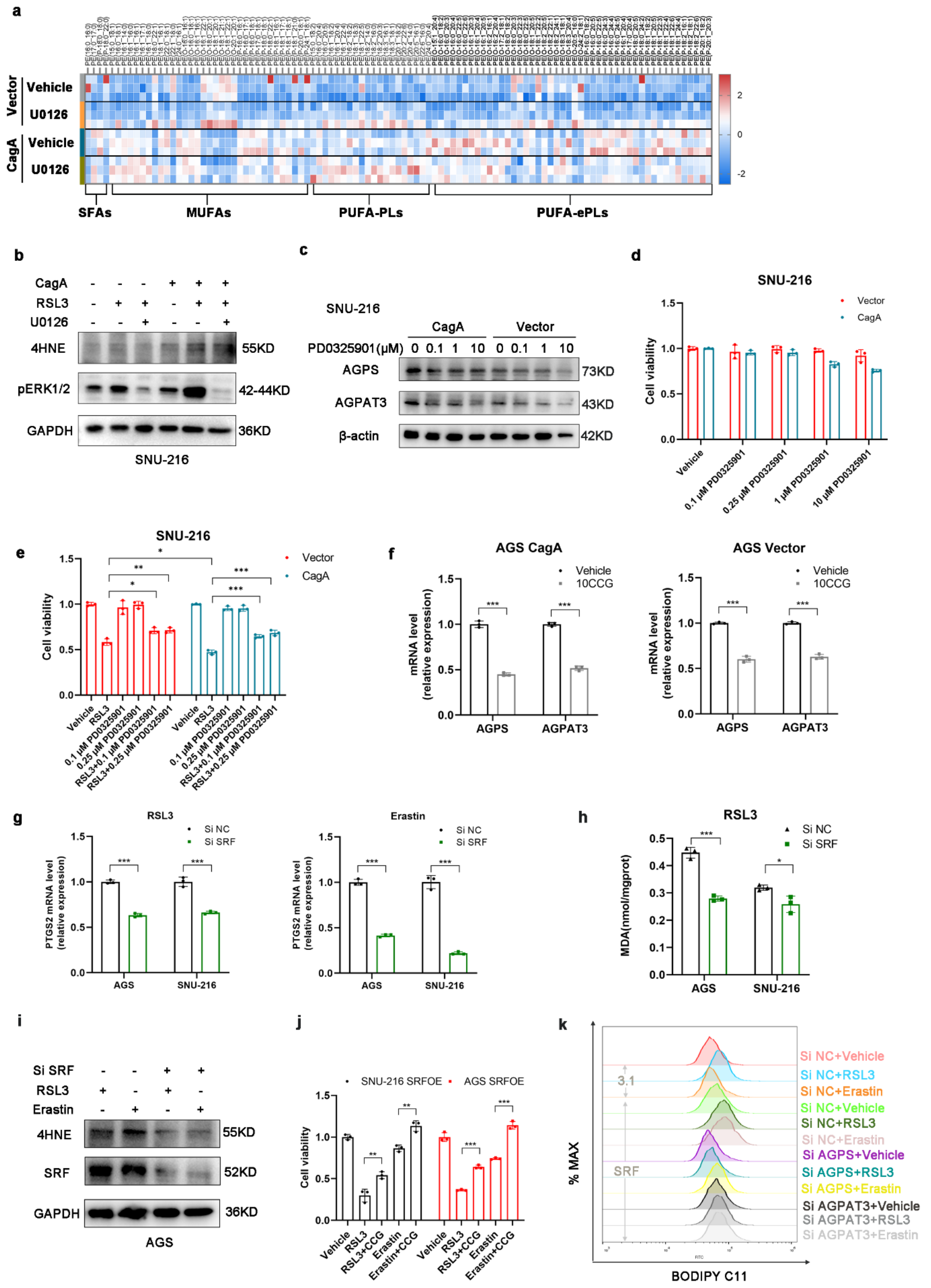
a
Vector
Vehicle
U0126
CagA
Vehicle
U0126
2
0
-2
SFAs
MUFAs
PUFA-PLs
PUFA-ePLs
b
CagA
RSL3
U0126
4HNE
55KD
pERK1/2
42-44KD
GAPDH
36KD
SNU-216
c
SNU-216
CagA
Vector
PD0325901(μM) 0 0.1 1 10 0 0.1 1 10
AGPS
73KD
AGPAT3
43KD
β-actin
42KD
d
SNU-216
Vector
CagA
Cell viability
Vehicle
0.1 μM PD0325901
0.25 μM PD0325901
1 μM PD0325901
10 μM PD0325901
e
SNU-216
Vector
CagA
Cell viability
Vehicle
RSL3
0.1 μM PD0325901
0.25 μM PD0325901
RSL3+0.1 μM PD0325901
RSL3+0.25 μM PD0325901
f
AGS CagA
AGS Vector
Vehicle
10CCG
mRNA level (relative expression)
AGPS
AGPAT3
g
RSL3
Erastin
Si NC
Si SRF
PTGS2 mRNA level (relative expression)
AGS
SNU-216
h
RSL3
Si NC
Si SRF
MDA(nmol/mgprot)
AGS
SNU-216
i
Si SRF
RSL3
Erastin
4HNE
55KD
SRF
52KD
GAPDH
36KD
AGS
j
SNU-216 SRFOE
AGS SRFOE
Cell viability
Vehicle
RSL3
RSL3+CCG
Erastin
Erastin+CCG
k
% MAX
3.1
SRF
Si NC+Vehicle
Si NC+RSL3
Si NC+Erastin
Si NC+Vehicle
Si NC+RSL3
Si NC+Erastin
Si AGPS+Vehicle
Si AGPS+RSL3
Si AGPS+Erastin
Si AGPAT3+Vehicle
Si AGPAT3+RSL3
Si AGPAT3+Erastin
BODIPY C11

Supplementary Fig. 3. CagA facilitates ferroptosis in GC cells via MEK/ERK/SRF-mediated ether lipids biosynthesis

(**a**) Heat map showing relative amounts of PE molecular species in SNU-216-CagA cells treated with U0126 (10 μM) for 24 h. Highlighted in bold are PUFA-containing ether lipids. (**b**) 4HNE and pERK1/2 protein levels detected by western blotting in indicated GC cells treated with vehicle or RSL3 (5 μM) with or without U0126 (10 μM) for 24 h. (**c**) Western blot analysis of AGPS and AGPAT3 expression in GC cells treated with PD0325901 for 24 h. (**d**) Relative cell viability of GC cells treated with PD0325901. (**e**) Relative viability of indicated GC cells treated with RSL3 with or without PD03225901 (0.1 μM and 0.25 μM) for 24 h. (**f**) AGPS and AGPAT3 expression after treated with CCG-1423 (10 μM) were assessed by qRT-PCR. (**g**) Fold changes in the expression of *PTGS2* following treatment with RSL3 (5 μM for AGS, 10 μM for SNU-216) or erastin (10 μM for AGS, 15 μM for SNU-216) in WT or SRF- knockdown GC cells. (**h**) Changes in cellular malondialdehyde levels in WT or SRF- knockdown GC cells treated with RSL3 (5 μM for AGS, 10 μM for SNU-216) for 12 h. (**i**) 4HNE and SRF protein levels detected by western blotting in indicated GC cells treated with RSL3 (5 μM) or erastin (10 μM) for 24 h. (**j**) Relative viability of SRF-overexpressing GC cells treated with RSL3 (5 μM for AGS, 15 μM for SNU-216) or erastin (20 μM) with or without CCG-1423 (10 μM) for 24 h. (**k**) Cell lipid peroxidation was detected by BODIPY 581/591 C11 staining using flow cytometry. Data represented as mean ± SD, *$p < 0.05$, **$p < 0.01$, ***$p < 0.001$.

Supplementary Table 1. The clinical-pathological characteristics of 42 clinical samples

Sample No#	Age	Gender	Borrmann Type	PTNM stage	CagA statue
1	67	Male	III	T3N1M0	-
2	58	Male	III	T4aN2M0	-
3	47	Male	IV	T4bN3bM1	-
4	61	Female	IV	T3N2M1	-
5	78	Male	III	T3N3aM0	+
6	62	Male	III	T3N3bM0	+
7	68	Female	III	T3N0M0	+
8	67	Female	IIc	T1aN0M0	+
9	34	Female	III	T3N2M0	-
10	38	Female	III	T4aN3aM0	+
11	80	Male	IV	T4aN3bM1	+
12	35	Female	III	T3N0M0	+
13	70	Male	I	T3 N3aM0	+
14	40	Female	III	T4aN3bM1	+
15	70	Male	IV	T4aN0M0	-
16	45	Male	III	T3N2M0	-
17	79	Male	III	T3N0M0	-
18	68	Male	I	T2N1M0	+
19	55	Male	III	T3N0M0	+
20	81	Male	I	T1bN0M0	-
21	63	Male	III	T4bN3bM1	+
22	63	Female	III	T1bN0M0	-
23	61	Male	III	T4aN3aM1	-
24	50	Male	III	T4aN3aM0	-
25	40	Female	IIc	T1aN0M0	+
26	70	Male	III	T4aN2Mx	-
27	77	Female	IV	T3N2M0	-
28	44	Female	III	T1bN1M0	-
29	56	Female	III	T3N0M0	-
30	58	Female	III	T3N2M0	-
31	36	Male	III	T2N2M0	-
32	69	Male	III	T4aN0M0	-
33	66	Male	III	T4aN3aM0	-
34	73	Male	III	T3N1M0	+
35	77	Female	III	T4aN3aM0	+
36	66	Male	III	T4aN1M1	+
37	60	Female	II	T2N0M0	+
38	60	Male	III	T2N1M0	+
39	51	Male	III	T4aN3aM0	-
40	35	Male	III	T3N0M0	-
41	56	Female	I	T2N1M0	-
42	63	Male	III	T3N1M0	-

Supplementary Table 2. Primers used for PCR

Gene	Primer	Nucleotide sequence (5’→3’)
CagA	Forward	GATAACAGGCAAGCTTTTGAGG
	Reverse	CTGCAAAAGATTGTTTGGCAGA
16S rRNA	Forward	GCGCAATCAGCGTCAGGTAATG
	Reverse	GCTAAGAGATCAGCCTATGTCC
ureA	Forward	GCCAATGGTAAATTAGTT
	Reverse	CTCCTTAATTGTTTTTAC

Supplementary Table 3. Primers used for qRT-PCR

Gene	Primer	Sequence (5'→3')
GNPAT	Forward	GAGGAGGCATGTCAGTGACTT
	Reverse	ACAAAACCGAATGGCTCCAAG
PEX3	Forward	TGCTTCCAACACTGAGAGAGG
	Reverse	AGAACAACCAGCATACAGGTACT
PEX7	Forward	TTGATGTGACTTGGAGTGAGAAC
	Reverse	CCCACAATTTGACAGTTTGATCC
PEX10	Forward	TCTGCTGGGAGTGCATCAC
	Reverse	CGAAGGTAGATGAGCTTCTGGG
AGPAT3	Forward	CTGCTGGTCGGCTTTGTCTT
	Reverse	TCCAGAGTGAGTAGGCGAGG
AGPS	Forward	TGAGTACCAATGAGTGCAAAGC
	Reverse	GGTAAACCCATGCCACTAAGAG
FAR1	Forward	AGACACCACAAGAGCGAGTG
	Reverse	CCAGTTTAGGTTGGGTGAGTTC
PEX12	Forward	CTTGCAGAATCAAATCCCACCC
	Reverse	GCCAATCTCTGAGACTTGTGAGT
PEX1	Forward	CATGCCGTAGTCAGGATAACTC
	Reverse	TGGTGGTAGTAGACTGCTGTAG
TMEM189	Forward	TCTGTGGAGCTGCCCATTG
	Reverse	GGTGAAGGTGCCGAAGATGA
PTGS2	Forward	CGGTGAAACTCTGGCTAGACAG
	Reverse	GCAAACCGTAGATGCTCAGGGA
ACSL4	Forward	GCTATCTCCTCAGACACACCGA
	Reverse	AGGTGCTCCAACTCTGCCAGTA
LPCAT3	Forward	GGCTGGATACTATTACACTGCC
	Reverse	GATCTTTCCCTCCGTCAAAGTAG
ELOVL5	Forward	AATAAACAGCCATTCTCTTGCC
	Reverse	GCCTTCCCATACTCCTGTTAC
FADS1	Forward	CAGACATCAACATGCATCCCT
	Reverse	AGAAGTATTTGTGCTGGTGGT
TMEM164	Forward	AAGTTCGCCACCAAGACCGTCA
	Reverse	GCTTGAAGACGACGATAGCTCC
β-actin	Forward	TGGCACCCAGCACAATGAA
	Reverse	CTAAGTCATAGTCCGCCTAGAAGCA

Supplementary Table 4. siRNAs for interference

siRNAs for AGPS interference		
siAGPS- 1	Sense	GCGAAUUCCUGAUAUAGUUTT
	Antisense	AACUAUAUCAGGAAUUCGCTT
siAGPS- 2	Sense	GAGUAAAUGUGGAGCAUAATT
	Antisense	UUAUGCUCCACAUUUACUCTT
siRNAs for AGPAT3 interference		
siAGPAT3- 1	Sense	GUCUGGUCAUCAACUUCGUTT
	Antisense	ACGAAGUUGAUGACCAGACTT
siAGPAT3- 2	Sense	GCAGCUCAGUGGCUUCAUATT
	Antisense	UAUGAAGCCACUGAGCUGCTT
siRNA for SRF interference		
siSRF	Sense	GGUGUUCCUGACAGCAUCATT
	Antisense	UGAUGCUGUCAGGAACACCTT
Negative control		
siNC	Sense	GGUUCUCCGAACGUGUCACGU
	Antisense	ACGUGACACGUUCGGAGAACC

Supplementary Table 5. Clinical information of the GC patients

Sample No#	Age	Gender	Borrmann Type	PTNM stage	CagA statue
01	58	Female	III	T3N2M0	-
02	77	Female	IV	T3N2M0	-
03	66	Male	III	T4aN3aM0	-
04	61	Male	III	T4aN3aM1	-
05	66	Male	III	T4aN1M1	+
06	63	Male	III	T4bN3bM1	+
07	77	Female	III	T4aN3aM0	+
